# Supplementary material for: Developmental decrease of entorhinal-hippocampal communication in immune-challenged DISC1 knockdown mice
Source: Nat Commun. 2021 Nov 23;12:6810. doi: 10.1038/s41467-021-27114-w (PMC8611076; doi:10.1038/s41467-021-27114-w)
Supplement: Supplementary file 1 — Supplementary Information [file 41467_2021_27114_MOESM1_ESM.pdf]

Supplementary information

Developmental decrease of entorhinal-hippocampal communication in immune-challenged DISC1 knockdown mice

Xu et.al

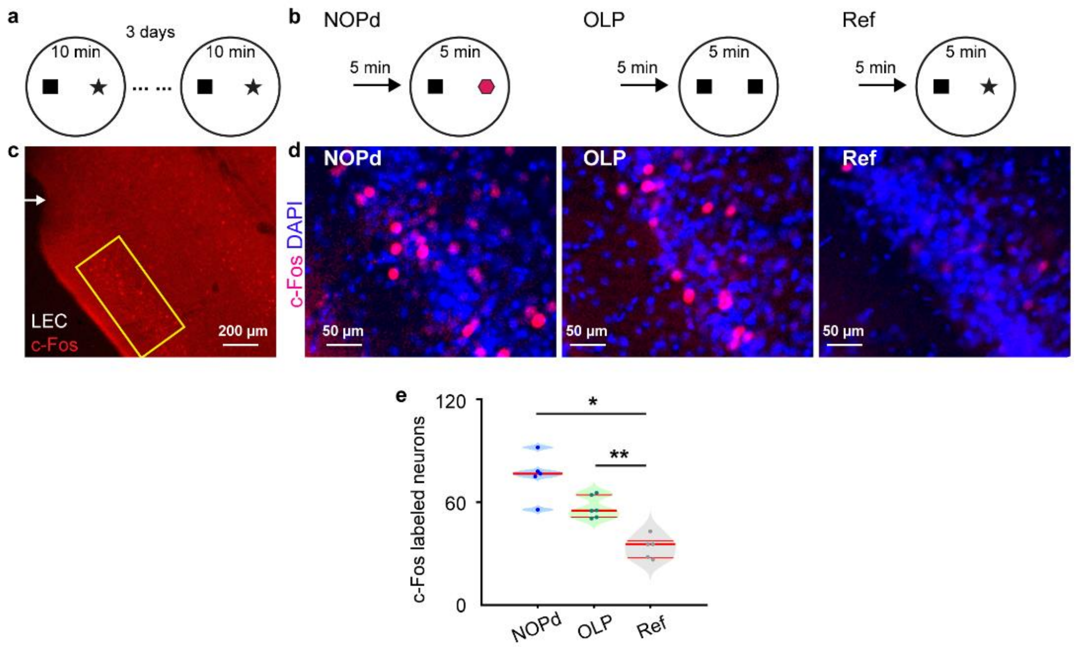

Supplementary fig.1 cFos immunoreactivity in LEC of mice tested in associative recognition tasks. (a) Schematic of the protocol for the familiarization trials (3 days, 2 trials per day). **(b)** Schematics of the protocol for NOPd test trial, OLP test trial, and reference trials (Ref). **(c)** Photomicrographs depicting cFos-expressing neurons (red) in the superficial layer of LEC from a P20 mouse, 90 mins after the test trial. **(d)** Photograph displaying the cFos-expressing cells (red dots) in LEC when stained for DAPI (blue) 90 mins after the NOPd test trial (left, n=5), OLP test trial (middle, n=6) and Ref trial (right, n=5). **(e)** Violin plots displaying the total number of cFos-positive neurons in the LEC of CON after NOPd task, OLP task, or Ref task (one-way ANOVA, p=2.35e-5, $F_{(2, 13)}$=26.92). For violin plots, black and red dots correspond to individual animals and the red horizontal lines display the median as well as 25th and 75th percentiles. *p<0.05, **p<0.01.

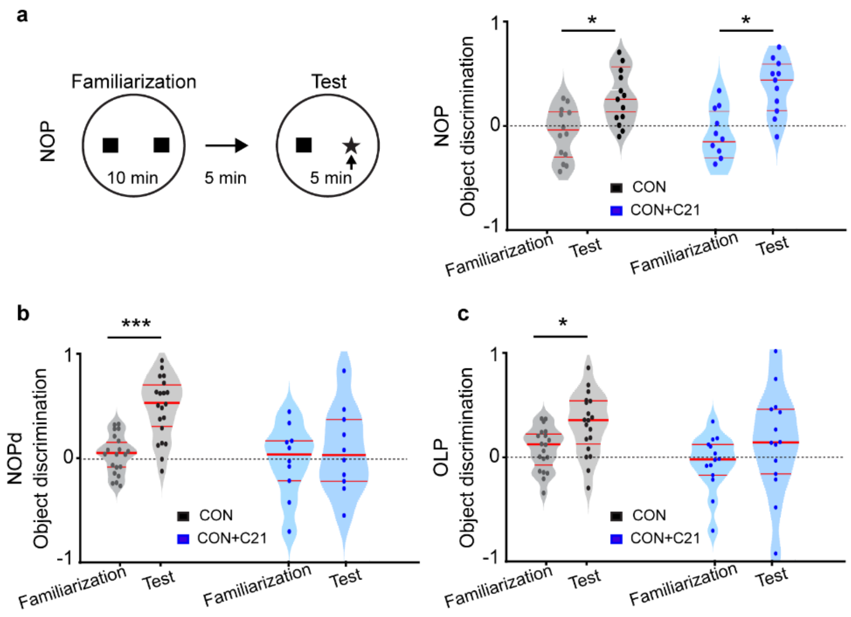

Supplementary fig.2 Behavioral performance of CON mice in NOP, NOPd, and OLP tasks after silencing of LEC activity by the DREADD agonist 21 (compound 21, C21). (a) Left, schematic of the protocol for NOP task. Right, violin plots displaying the discrimination ratio in familiarization and test trials of NOP task (paired-sample t-test, CON, p=0.04, df=15, t=-1.87; CON+C21: p=0.02, df=9, t=-2.65). **(b)** Violin plots displaying the discrimination ratio in familiarization and test trials of NOPd task (paired-sample t-test, p=1.2e-5, df=18, t=-5.54 for CON mice). **(c)** Same as (b) for OLP task (paired-sample t-test, p=0.014, df=18, t=-2.69 for CON mice). Black and red dots correspond to individual animals and the red horizontal lines display the median as well as 25th and 75th percentiles.

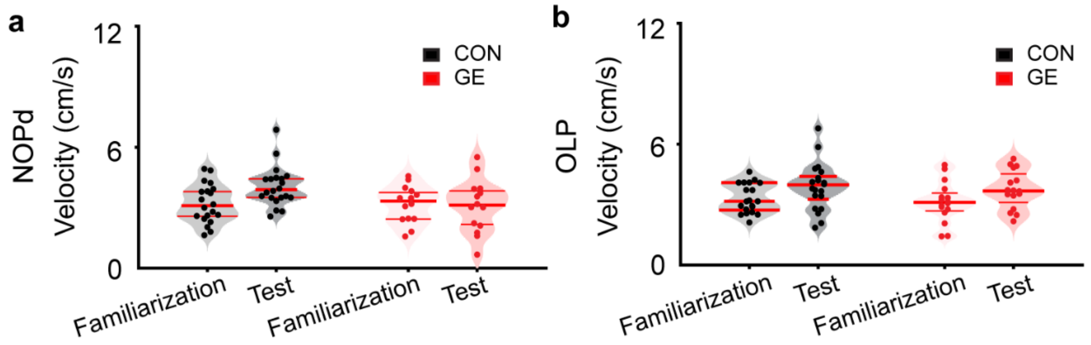

Supplementary fig.3 The velocity of pre-juvenile CON and GE mice in NOPd and OLP tasks. (a) Violin plots displaying the exploration velocity in familiarization and test trials of NOPd tasks. **(b)** The same display as (a) for OLP task. Black and red dots correspond to individual animals and the red horizontal lines display the median as well as 25th and 75th percentiles.

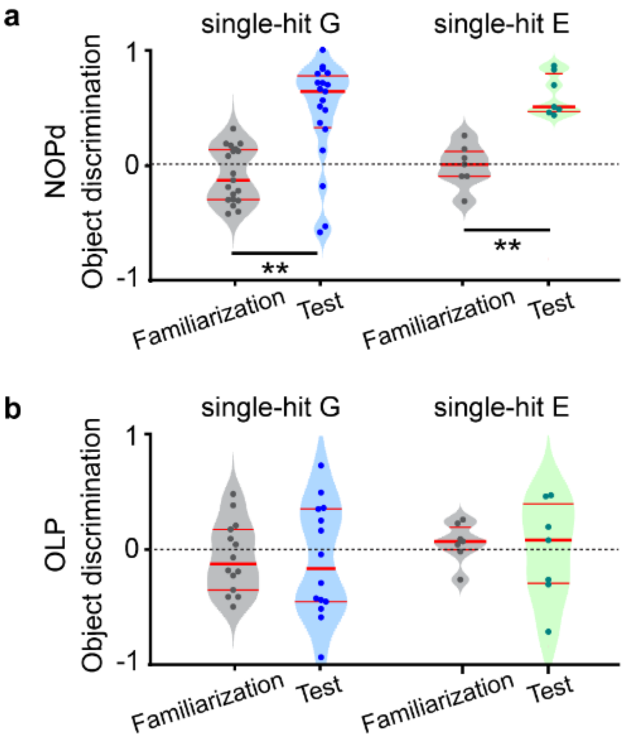

Supplementary fig.4 The performance of pre-juvenile single-hit G and E mice in associative recognition memory tasks. (a) Violin plots displaying the discrimination ratio in familiarization and test trials when averaged for single-hit G (DISC1 mice, paired-sample t-test, p=0.0016, df=18, t=-3.69) and E (poly I:C-treated dams to induce MIA, paired-sample t-test, p=0.003, df=6, t=-4.70) mice. The black dotted line indicates chance level. **(b)** The same display as (a) for OLP task. The black dotted line indicates chance level. Black and red dots correspond to individual animals and the red horizontal lines display the median as well as 25th and 75th percentiles. **p<0.01.

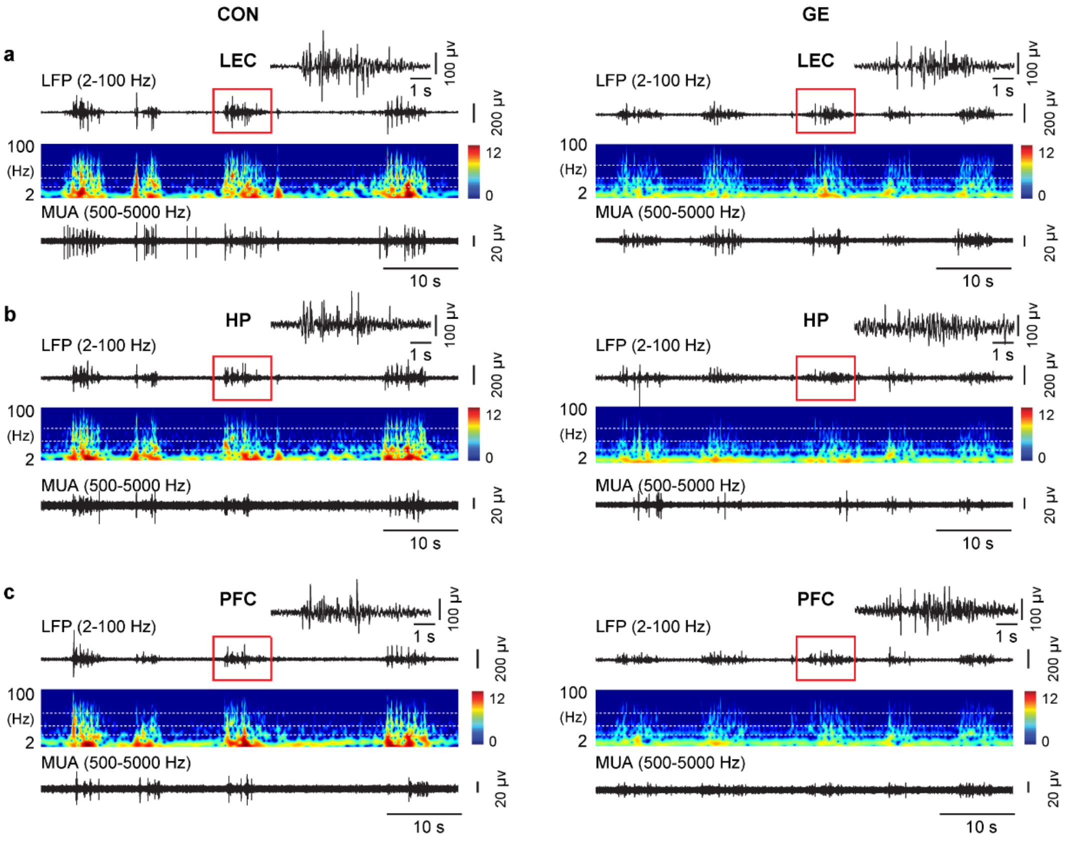

Supplementary fig.5 Discontinuous network activity in the LEC, HP and PFC of neonatal CON and GE mice. (a) Extracellular LFP recordings of discontinuous oscillatory activity in the LEC of a P9 CON mouse (left) and a P9 GE mouse (right) displayed after bandpass (2-100 Hz) filtering (top) together with the corresponding MUA (500-5000 Hz) (bottom). Traces are accompanied by the color-coded wavelet spectra of the LFP at identical time scale. **(b, c)** Same as (a) for HP and PFC, respectively.

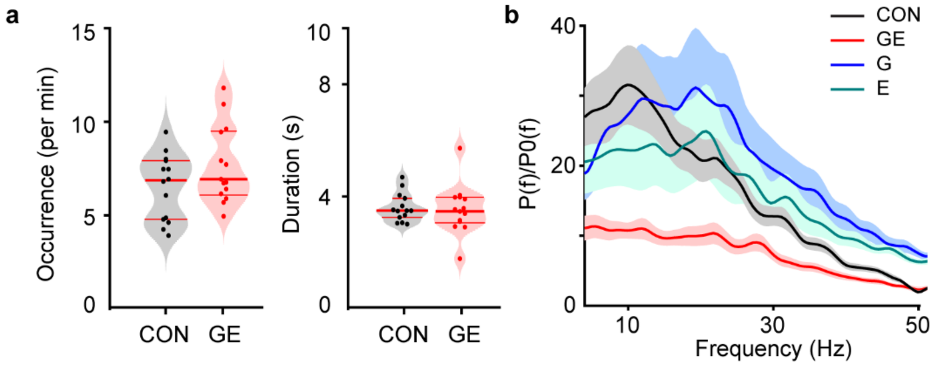

Supplementary fig.6 Patterns of network activity in the LEC of neonatal CON, GE, G, and E mice. (a) Violin plots displaying the occurrence and the duration of oscillatory activity in LEC recorded in CON and GE mice. **(b)** Averaged power spectra P(f) of discontinuous oscillatory activity normalized to the baseline power P0(f) of time windows lacking oscillatory activity in CON, double-hit GE, single-hit G and single-hit E mice. For violin plots, black and red dots correspond to individual animals and the red horizontal lines display the median as well as 25th and 75th percentiles.

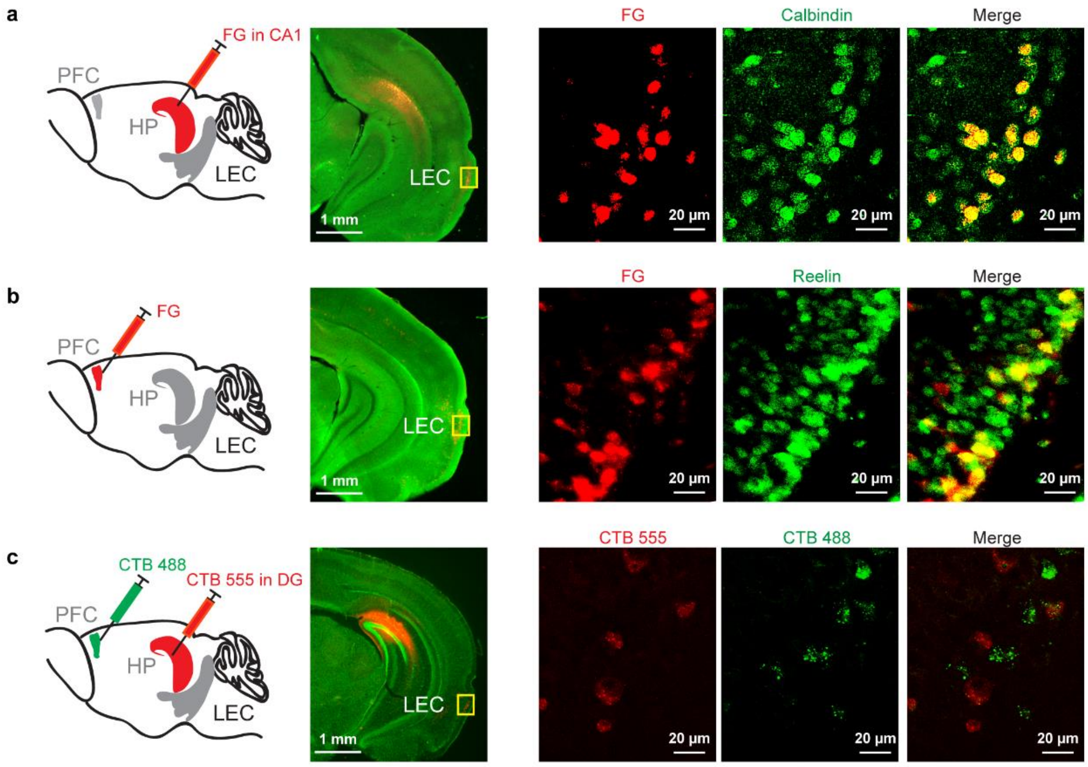

Supplementary fig.7 Co-localization of CA1-projecting neurons with reelin, and PFC-projecting neurons with calbindin in LEC. (a) Schematic of the retrograde FG injection in HP. Middle, photograph depicting FG-labeled neurons in the LEC of a P10 CON mouse (total n=3 mice). Right, FG and calbindin-labeled neurons in LEC. **(b)** Schematic of the retrograde FG injection in PFC. Middle, photograph depicting FG-labeled neurons in the LEC of a P10 CON mouse (total n=3 mice). Right, FG and reelin-labeled neurons in LEC. **(c)** Schematic of the retrograde CTB 488 injection in PFC, CTB 555 injection in DG. Middle, photograph depicting CTB 555-labeled neurons in the LEC of a P10 CON mouse (total n=2 mice). Right, CTB 488-labeled neurons and CTB 555-labeled neurons in the LEC.

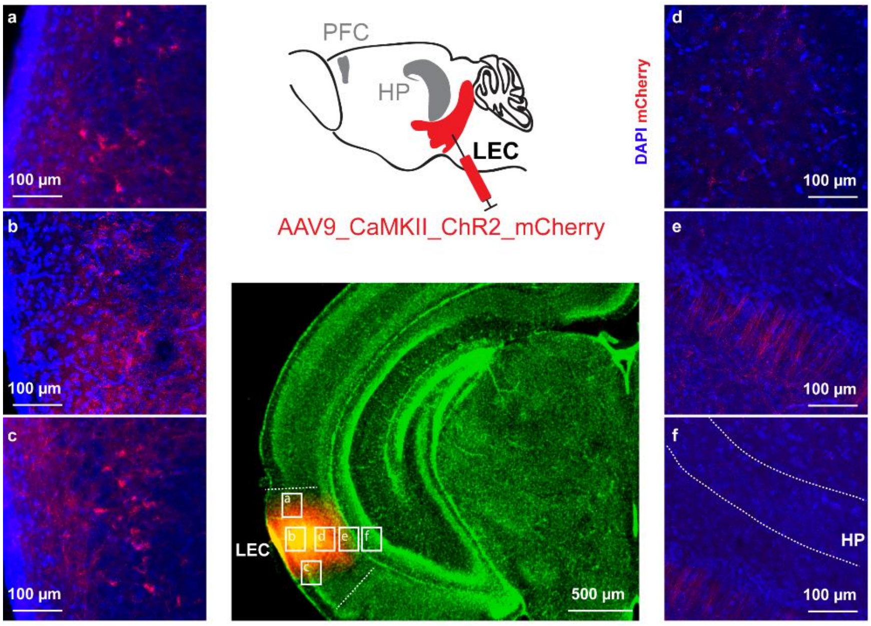

Supplementary fig.8 AAV9-CaMKII-ChR2-mCherry expression in LEC. AAV9-CaMKII-ChR2-mCherry injection in the LEC (middle, total n=12 mice) led to mCherry-labeled neurons in superficial layer of LEC **(a, b, c)**, deep layer of LEC **(d)**, axons from LEC to HP **(e)** and no transfected neurons in HP **(f)**.

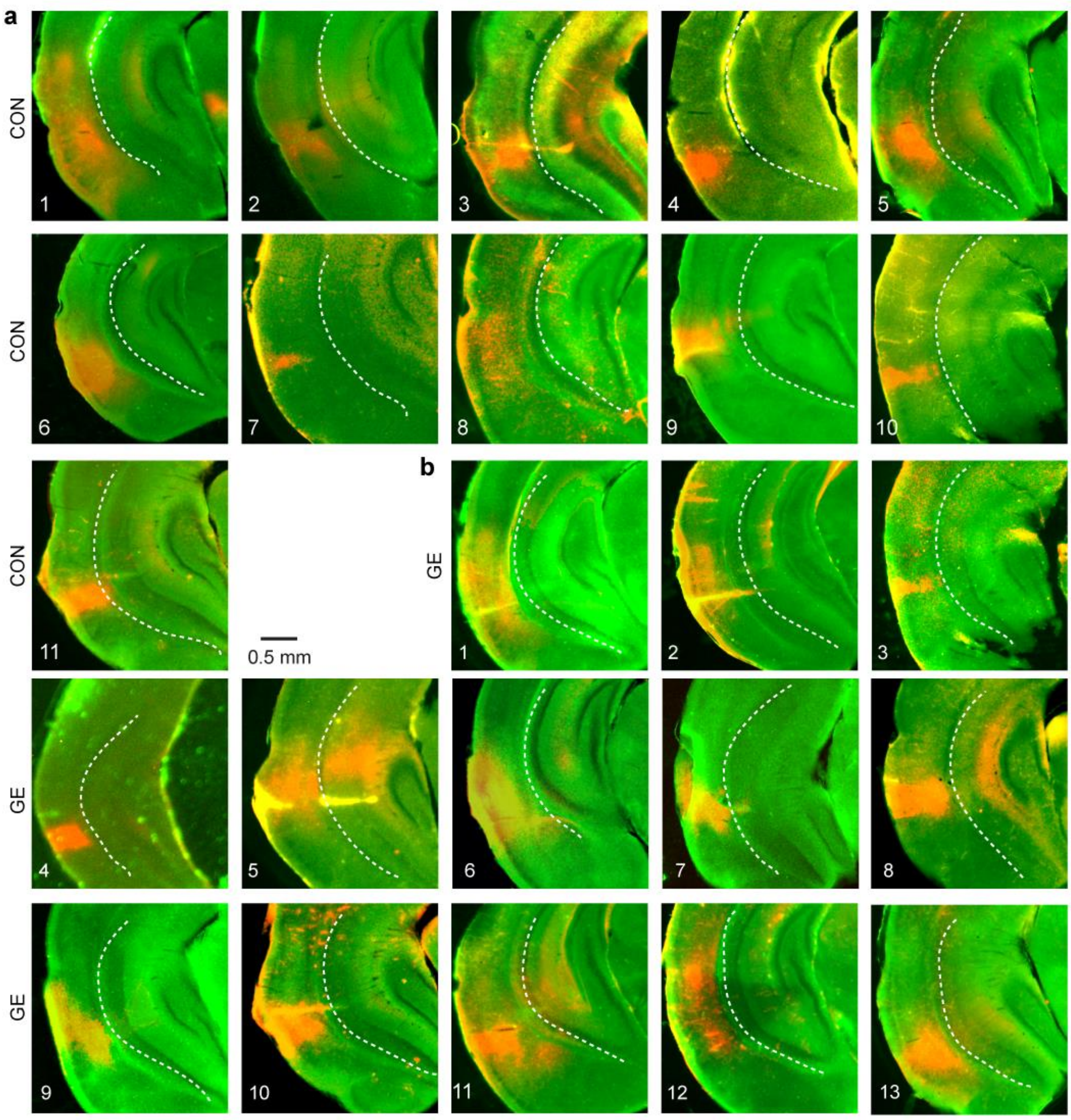

Supplementary fig.9 The injection position of AAV9-CaMKII-ChR2-mCherry in LEC for all the mice. (a) Photographs depicting the injection position for all CON mice used in the study. **(b)** Photographs depicting the injection position for all GE mice used in the study.

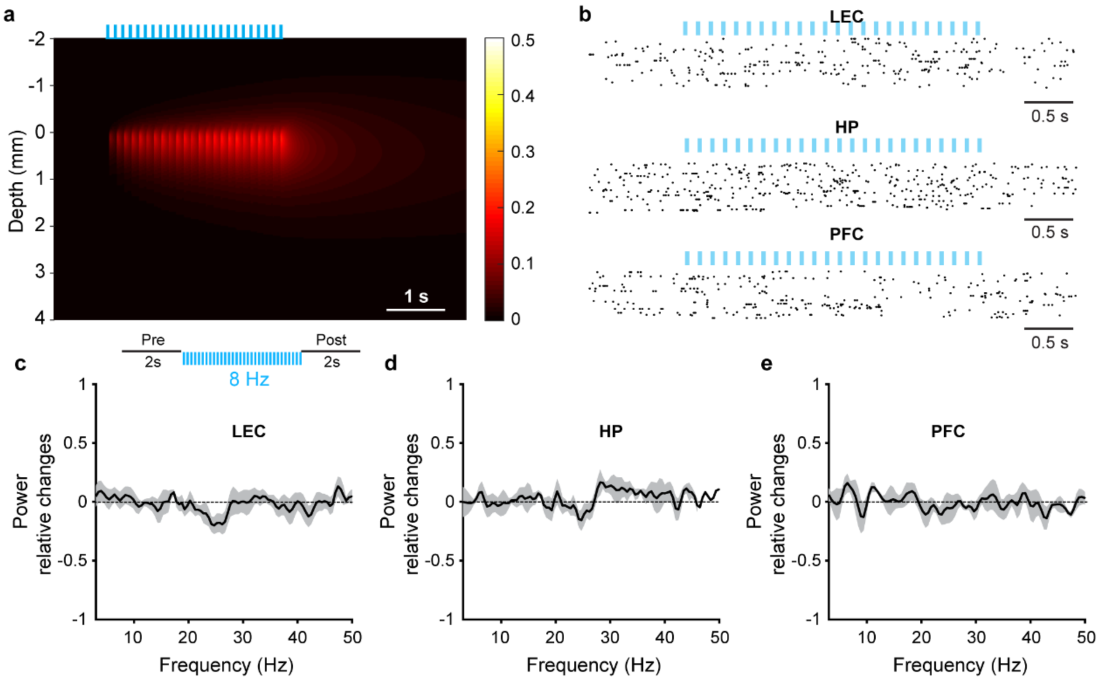

Supplementary fig.10 Pulses stimulation did not affect the firing activity and oscillatory power in opsin-free CON mice. (a) Heat map of temperature changes caused by the 8 Hz pulse stimulation estimated by the Monte Carlo model. **(b)** Representative raster plots of LEC firing activity in response to 30 sweeps of 8 Hz-pulsed stimulation (3 ms pulse length, 473 nm) in LEC (up), HP (middle) and PFC (bottom) of one P9 CON mice. **(c)** Line plot depicting the power of entorhinal oscillatory activity after stimulation of LEC (post) normalized to the activity before the stimulation (pre). **(d, e)** Same display as **(c)** for HP and PFC, respectively.

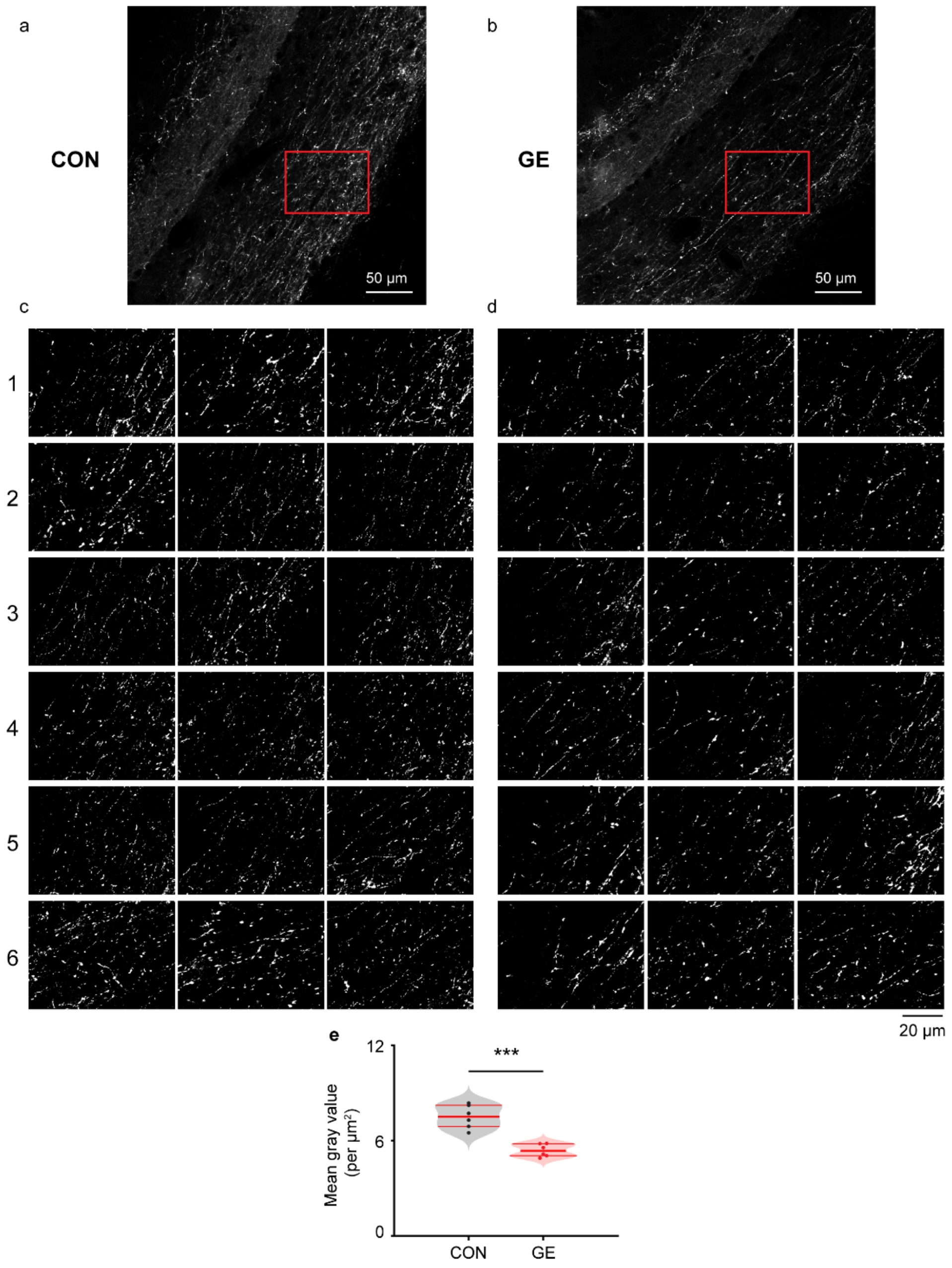

Supplementary fig.11 LEC axons labeled by mCherry in HP. (a, b) Photographs exemplarily illustrating entorhinal axons in the HP of a P10 CON (a, n=6) and a GE mouse (b, n=6). **(c)** Axons from the area marked by a red box shown at higher-magnification. Totally 6 CON mice (each line indicated one mouse, each image indicated one slice were used for the quantification. **(d)** Same display as (c) for 6 GE mice. **(e)** Violin plot displaying the averaged intensity of mCherry in CON and GE mice (one-way ANOVA, p=1.09e-4, $F_{(1,\ 10)}$=37.76). For violin plots, black and red dots correspond to individual animals and the red horizontal lines display the median as well as 25th and 75th percentiles. ***p<0.0001.

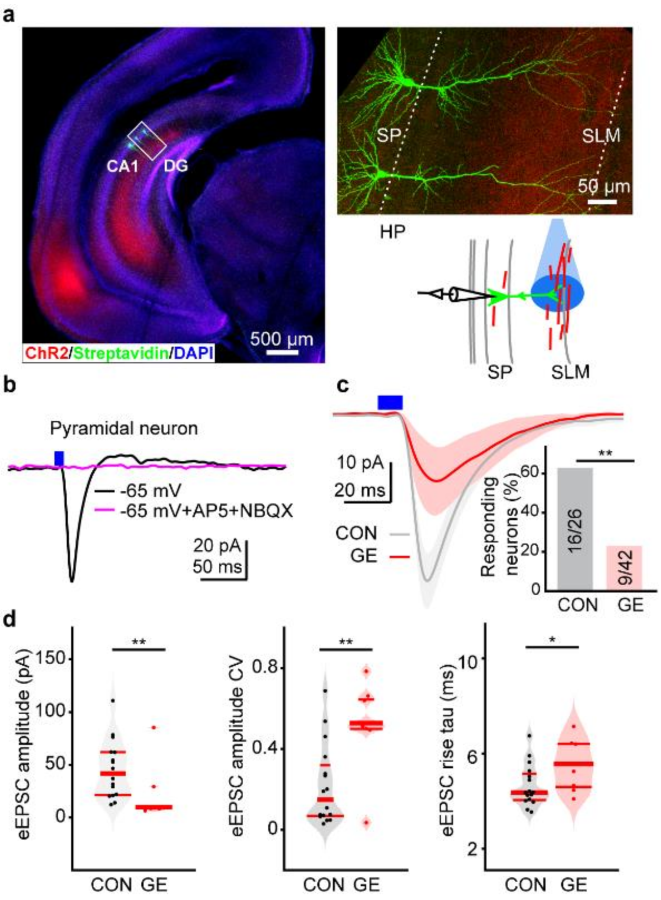

Supplementary fig.12 Synaptic properties of entorhinal inputs on CA1 pyramidal neurons in neonatal CON and GE mice. **(a)** Left, representative image showing ChR2 (H134R) (red) expression in a DAPI-stained coronal slice from a P10 CON mouse following LEC transfection at P1. Right, confocal image showing biocytin-filled CA1 neurons in HP from a P10 CON mouse displayed together with a schematic of light stimulation / recording protocol (total n= 4 mice). **(b)** Representative light-evoked responses (blue vertical bar, 10 ms) recorded in a CA1 pyramidal neuron (black trace) at -65 mV. The response was abolished by bath application of NBQX and AP5 (purple trace). **(c)** Average light-evoked responses HP neurons from CON (n=16) and GE (n=9) neurons. Inset, bar diagram of the percentage of responsive CA1 neurons (p=0.0021, chi=9.45, Chi-square test). **(d)** Violin plots displaying the amplitude (left, Wilcoxon rank-sum test, p=0.0042, zval=2.86, ranksum=259), coefficient of variation of amplitudes (middle, one-way ANOVA, p=0.0016, $F_{(1, 23)}$=12.81), and rise time (right, one-way ANOVA, p= 0.036, $F_{(1, 23)}$=4.98) of light-evoked responses averaged for all CA1 neurons from CON (n=16) and GE (n=9) neurons. For violin plots, black and red dots correspond to individual investigated neurons and the red horizontal lines display the median as well as 25th and 75th percentiles. *p < 0.05, **p < 0.01.

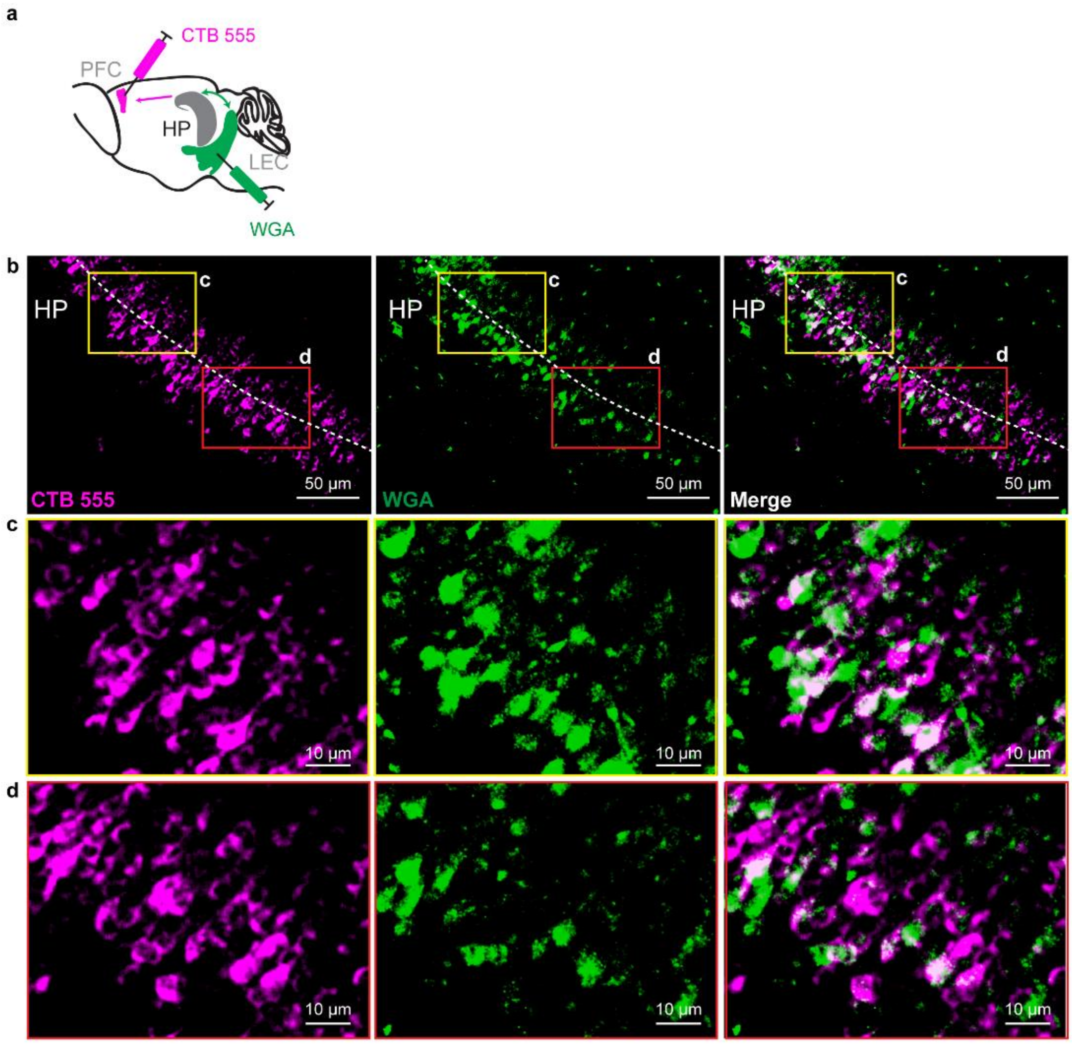

Supplementary fig.13 LEC-PFC communication achieved by bi-synaptic and poly-synaptic transmission through HP neurons. (a) Schematic of the retrograde CTB 555 injection in PFC and retro-/anterograde WGA injection in LEC. **(b)** Photograph depicting CTB-labeled and WGA-labeled neurons in the CA1 of a P10 CON mouse (total n=4 mice). **(c, d)** Photograph depicting the labeled CA1 neurons from the area marked by yellow and red boxes in (b) at higher-magnification.

Experiments	Interested region	Slice thickness	Slice collection	Mice number
**CTB in HP**	LEC	100 μm	Three equally spaced series, 3 slices	CON, 5<br>GE, 6
**CTB in PFC**	LEC			CON, 6<br>GE,5
**AAV9_ mCherry in LEC**	HP			CON, 6<br>GE,5
**cFos expression**	LEC	50 μm	Six equally spaced series, 3 slices	NOPd, 5<br>OLP, 6<br>Ref, 5

Supplementary table 1. Slice selection for quantification.
