# Supplementary material for: Developmental decrease of entorhinal-hippocampal communication in immune-challenged DISC1 knockdown mice
Source: Nat Commun. 2021 Nov 23;12:6810. doi: 10.1038/s41467-021-27114-w (PMC8611076; doi:10.1038/s41467-021-27114-w)
Supplement: Supplementary file 3 — Reporting Summary [file 41467_2021_27114_MOESM3_ESM.pdf]

nature research

Corresponding author(s): Ileana Hanganu-Opatz

Last updated by author(s): 13/10/2021

Reporting Summary

Nature Research wishes to improve the reproducibility of the work that we publish. This form provides structure for consistency and transparency in reporting. For further information on Nature Research policies, see our Editorial Policies and the Editorial Policy Checklist.

Statistics

For all statistical analyses, confirm that the following items are present in the figure legend, table legend, main text, or Methods section.

n/a	Confirmed	
☐	☒	The exact sample size (*n*) for each experimental group/condition, given as a discrete number and unit of measurement
☐	☒	A statement on whether measurements were taken from distinct samples or whether the same sample was measured repeatedly
☐	☒	The statistical test(s) used AND whether they are one- or two-sided<br>*Only common tests should be described solely by name; describe more complex techniques in the Methods section.*
☐	☒	A description of all covariates tested
☐	☒	A description of any assumptions or corrections, such as tests of normality and adjustment for multiple comparisons
☐	☒	A full description of the statistical parameters including central tendency (e.g. means) or other basic estimates (e.g. regression coefficient) AND variation (e.g. standard deviation) or associated estimates of uncertainty (e.g. confidence intervals)
☐	☒	For null hypothesis testing, the test statistic (e.g. *F*, *t*, *r*) with confidence intervals, effect sizes, degrees of freedom and *P* value noted<br>*Give P values as exact values whenever suitable.*
☐	☒	For Bayesian analysis, information on the choice of priors and Markov chain Monte Carlo settings
☒	☐	For hierarchical and complex designs, identification of the appropriate level for tests and full reporting of outcomes
☒	☐	Estimates of effect sizes (e.g. Cohen's *d*, Pearson's *r*), indicating how they were calculated

Our web collection on statistics for biologists contains articles on many of the points above.

Software and code

Policy information about availability of computer code

Data collection	Digital Lynx 4SX(https://neuralynx.com/), Cheetah 6 (https://neuralynx.com/), Video Mot2(https://www.tse-systems.com/product-details/videomot)
Data analysis	https://github.com/XiaxiaXu/Toolbox_LFP_Spike

For manuscripts utilizing custom algorithms or software that are central to the research but not yet described in published literature, software must be made available to editors and reviewers. We strongly encourage code deposition in a community repository (e.g. GitHub). See the Nature Research guidelines for submitting code & software for further information.

Data

Policy information about availability of data

All manuscripts must include a data availability statement. This statement should provide the following information, where applicable:

- Accession codes, unique identifiers, or web links for publicly available datasets
- A list of figures that have associated raw data
- A description of any restrictions on data availability

LFP and MUA data for all recordings are available at the following open-access repository: https://gin.g-node.org/xiaxiaxu/Developmental_ephys_LEC-HP-PFC/. Further data supporting the findings of this study are available from the corresponding authors on request.

Field-specific reporting

Please select the one below that is the best fit for your research. If you are not sure, read the appropriate sections before making your selection.

☒ Life sciences ☐ Behavioural & social sciences ☐ Ecological, evolutionary & environmental sciences

For a reference copy of the document with all sections, see nature.com/documents/nr-reporting-summary-flat.pdf

Life sciences study design

All studies must disclose on these points even when the disclosure is negative.

Sample size	The sample size is decided by calculate the variance of the results. If the variance was big, we added more number of the mice. To the end, the number of mice used for each experiments was between 10 to 20.
Data exclusions	No data exclusions except for the result of Fig.7c. For Fig.7c, 1.5IQR rule was used (https://en.wikipedia.org/wiki/Interquartile_range).
Replication	To make results reproducible, we strongly controlled the process of data acquisition, data processing and data analysis. For example, all recording data are clearly labeled and documented. The details of each operations related to data acquisition were also documented. The code used for data analysis was also documented, etc. By doing so, the results in this project are replication.
Randomization	Mice were randomly allocated into two groups.
Blinding	The investigators were blinded to group allocation during data collection and analysis.

Reporting for specific materials, systems and methods

We require information from authors about some types of materials, experimental systems and methods used in many studies. Here, indicate whether each material, system or method listed is relevant to your study. If you are not sure if a list item applies to your research, read the appropriate section before selecting a response.

Materials & experimental systems

n/a	Involved in the study	
☐	☒	Antibodies
☒	☐	Eukaryotic cell lines
☒	☐	Palaeontology and archaeology
☐	☒	Animals and other organisms
☒	☐	Human research participants
☒	☐	Clinical data
☒	☐	Dual use research of concern

Methods

n/a	Involved in the study	
☒	☐	ChIP-seq
☒	☐	Flow cytometry
☒	☐	MRI-based neuroimaging

Antibodies

Antibodies used	Alexa Fluor-488 goat anti-rabbit IgG secondary antibody (AB_143165, Merck Millipore), rabbit anti-cFos (MA5-15055, ThermoFisher), rabbit anti lectin (A2052, Sigma-Aldrich)
Validation	First, we note the validation statements on the website of Sigma, Merck Millipore, ThermoFisher for the antibodies used. Second, we also checked the statement of the antibodies in related citations. Last, we tested each antibody on control mice. When the antibody was validated, we used it in the real experiment.

Animals and other organisms

Policy information about studies involving animals; ARRIVE guidelines recommended for reporting animal research

Laboratory animals	Timed-pregnant mice from the animal facility of the University Medical Center Hamburg-Eppendorf were housed individually at a 12 h light/12h dark cycle and were given access to water and food ad libitum. The temperature is about 23 degree and the humidity is about 56%.
Wild animals	The study did not involve wild animals.
Field-collected samples	The study did not involve samples collected from field.
Ethics oversight	All experiments were performed in compliance with the German laws and the guidelines of the European Community for the use of animals in research and were approved by the local ethical committee (015/17, 015/18).

Note that full information on the approval of the study protocol must also be provided in the manuscript.
